# 4-Hydroxy­benzoic acid–1*H*-imidazole (1/1)

**DOI:** 10.1107/S1600536809016043

**Published:** 2009-05-07

**Authors:** Wei Wang, Bang-Wei Liu, Jing Liu, Rui Ren

**Affiliations:** a College of Life Science and Pharmaceutical Engineering, Nanjing University of Technology, 210009 Nanjing, Jiangsu, People’s Republic of China; b College of Chemistry and Molecular Engineering, Qingdao University of Science and Technology, 266042 Qingdao, Shandong, People’s Republic of China

## Abstract

In the title 1:1 adduct, C_7_H_6_O_3_·C_3_H_4_N_2_, the crystal packing features π–π stacking inter­actions [centroid–centroid distances = 3.799 (2) and 3.753 (1) Å] as well as N—H⋯(O,O) O—H⋯O and C—H⋯O hydrogen bonds.

## Related literature

For related structures, see: Li *et al.* (2005 ▶); Wan *et al.* (2005 ▶). For the synthesis, see: Wang *et al.* (2006 ▶). For bond-length data, see Allen *et al.* (1987 ▶).
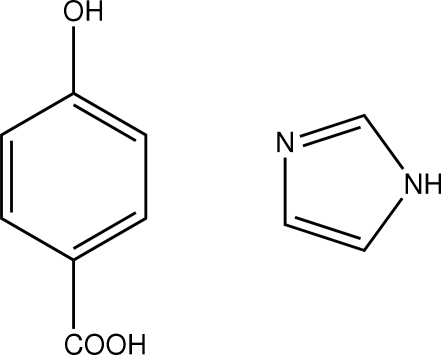


## Experimental

### 

#### Crystal data

C_7_H_6_O_3_·C_3_H_4_N_2_
*M* *_r_* = 206.20
Monoclinic, 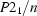

*a* = 9.601 (2) Å
*b* = 10.530 (2) Å
*c* = 10.586 (2) Å
β = 113.759 (3)°
*V* = 979.6 (4) Å^3^
*Z* = 4
Mo *K*α radiation
μ = 0.11 mm^−1^
*T* = 293 K
0.47 × 0.29 × 0.10 mm

#### Data collection

Siemens SMART 1000 CCD area-detector diffractometer
Absorption correction: multi-scan (*SADABS*; Sheldrick, 1996 ▶) *T*_min_ = 0.955, *T*_max_ = 0.987
5200 measured reflections
1858 independent reflections
1583 reflections with *I* > 2σ(*I*)
*R*_int_ = 0.024

#### Refinement

*R*[*F* ^2^ > 2σ(*F* ^2^)] = 0.066
*wR*(*F* ^2^) = 0.213
*S* = 1.11
1858 reflections
136 parameters
H-atom parameters constrained
Δρ_max_ = 0.61 e Å^−3^
Δρ_min_ = −0.47 e Å^−3^

### 

Data collection: *SMART* (Siemens, 1996 ▶); cell refinement: *SAINT* (Siemens, 1996 ▶); data reduction: *SAINT*; program(s) used to solve structure: *SHELXS97* (Sheldrick, 2008 ▶); program(s) used to refine structure: *SHELXL97* (Sheldrick, 2008 ▶); molecular graphics: *SHELXTL* (Sheldrick, 2008 ▶); software used to prepare material for publication: *SHELXTL*, *PARST* (Nardelli, 1995 ▶) and *PLATON* (Spek, 2009 ▶).

## Supplementary Material

Crystal structure: contains datablocks global, I. DOI: 10.1107/S1600536809016043/at2768sup1.cif

Structure factors: contains datablocks I. DOI: 10.1107/S1600536809016043/at2768Isup2.hkl

Additional supplementary materials:  crystallographic information; 3D view; checkCIF report

## Figures and Tables

**Table 1:** Hydrogen-bond geometry (Å, °)

*D*—H⋯*A*	*D*—H	H⋯*A*	*D*⋯*A*	*D*—H⋯*A*
N1—H1⋯O1	0.86	2.53	3.057 (3)	121
N1—H1⋯O2	0.86	1.82	2.678 (3)	177
O3—H3⋯O1^i^	0.82	1.83	2.635 (3)	166
C8—H8⋯O1^ii^	0.93	1.89	2.748 (3)	153

Symmetry codes: (i) 
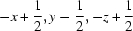
; (ii) 
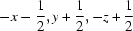
.

**Fig. 1.**
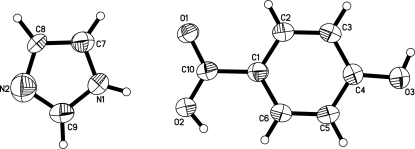
The structure of the compound (I) showing 50% probability displacement ellipsoids and the atom numbering scheme.

## supplementary crystallographic information

### Comment

Imidazole compounds have received considerable attention in the literature. We have reported the structure of 3-(1*H*-imidazol-1-yl)-1-phenylpropan-1-one, (II) (Li *et al.*, 2005). In order to obtain comprehensive structural information of imidazole compounds and in our ongoing search for new imidazole compounds, the title compound, (I), was prepared hydrothermally and we report its structure here.

A view of the molecule of the title compound, (I), is shown in Fig. 1. The bond lengths and angles are within normal ranges (Allen *et al.*, 1987). The bonds in the imidazole and hydroxybenzoate systems show intermediate character between single and double bonds, indicating a highly π-conjugated delocalization. The crystal structure is stabilized by π–π interactions involving the imidazole and hydroxybenzoate rings: *Cg*1···*Cg*1 (-*x*, 2 - *y*, 1 - *z*) = 3.799 Å and *Cg*1···*Cg*2 (-*x*, 1 - *y*, 1 - *z*) = 3.753 Å, where *Cg*1 and *Cg*2 denote the centroids of the N1/N2/C7—C9 imidazole and C1—C6 benzene rings, respectively. In the crystal packing, molecules are linked into three-dimension network by C—H···O and O—H···O intermolecular hydrogen bonds (Table 1).

### Experimental

The title compound was prepared according to the literature method of Wang *et al.* (2006). It was hydrothermally prepared from a reaction mixture of CdCl_2_.2.5H_2_O, 4-hydroxybenzoic acid, 1*H*-imidazole, and distilled water (10 ml) in a molar ratio of 1:2:6:555. The mixture was stirred for 20 min at room temperature and then crystallized in a Teflon-lined stainless steel autoclave with a 23 ml capacity at 433 K for five days. After cooling, single crystals of (I) suitable for X-ray measurements were obtained.

### Refinement

All H atoms were located in difference Fourier maps and constrained to ride on their parent atoms, with C—H = 0.93–0.96 Å, O—H = 0.82 Å and N—H = 0.86 Å, and with *U*_iso_(H) = 1.2 *U*_eq_(C,N) or 1.5 *U*_eq_(O) for hydroxy H atoms.

### Crystal data

**Table:**

C_7_H_6_O_3_·C_3_H_4_N_2_	*F*(000) = 440
*M**_r_* = 206.20	*D*_x_ = 1.398 Mg m^−^^3^
Monoclinic, *P*2_1_/*n*	Mo *K*α radiation, λ = 0.71073 Å
Hall symbol: -P 2yn	Cell parameters from 2125 reflections
*a* = 9.601 (2) Å	θ = 2.9–25.6°
*b* = 10.530 (2) Å	µ = 0.11 mm^−^^1^
*c* = 10.586 (2) Å	*T* = 293 K
β = 113.759 (3)°	Block, colourless
*V* = 979.6 (4) Å^3^	0.47 × 0.29 × 0.10 mm
*Z* = 4	

### Data collection

**Table:**

Siemens SMART 1000 CCD area-detector diffractometer	1858 independent reflections
Radiation source: fine-focus sealed tube	1583 reflections with *I* > 2σ(*I*)
graphite	*R*_int_ = 0.024
Detector resolution: 8.33 pixels mm^-1^	θ_max_ = 25.7°, θ_min_ = 2.4°
ω scans	*h* = −10→11
Absorption correction: multi-scan (*SADABS*; Sheldrick, 1996)	*k* = −12→12
*T*_min_ = 0.955, *T*_max_ = 0.987	*l* = −12→5
5200 measured reflections	

### Refinement

**Table:**

Refinement on *F*^2^	Primary atom site location: structure-invariant direct methods
Least-squares matrix: full	Secondary atom site location: difference Fourier map
*R*[*F*^2^ > 2σ(*F*^2^)] = 0.066	Hydrogen site location: inferred from neighbouring sites
*wR*(*F*^2^) = 0.213	H-atom parameters constrained
*S* = 1.11	*w* = 1/[σ^2^(*F*_o_^2^) + (0.1212*P*)^2^ + 0.6043*P*] where *P* = (*F*_o_^2^ + 2*F*_c_^2^)/3
1858 reflections	(Δ/σ)_max_ < 0.001
136 parameters	Δρ_max_ = 0.61 e Å^−^^3^
0 restraints	Δρ_min_ = −0.47 e Å^−^^3^

### Special details

**Table:**

Geometry. All e.s.d.'s (except the e.s.d. in the dihedral angle between two l.s. planes) are estimated using the full covariance matrix. The cell e.s.d.'s are taken into account individually in the estimation of e.s.d.'s in distances, angles and torsion angles; correlations between e.s.d.'s in cell parameters are only used when they are defined by crystal symmetry. An approximate (isotropic) treatment of cell e.s.d.'s is used for estimating e.s.d.'s involving l.s. planes.
Refinement. Refinement of *F*^2^ against ALL reflections. The weighted *R*-factor *wR* and goodness of fit *S* are based on *F*^2^, conventional *R*-factors *R* are based on *F*, with *F* set to zero for negative *F*^2^. The threshold expression of *F*^2^ > σ(*F*^2^) is used only for calculating *R*-factors(gt) *etc*. and is not relevant to the choice of reflections for refinement. *R*-factors based on *F*^2^ are statistically about twice as large as those based on *F*, and *R*- factors based on ALL data will be even larger.

### Fractional atomic coordinates and isotropic or equivalent isotropic displacement parameters (Å^2^)

**Table:**

	*x*	*y*	*z*	*U*_iso_*/*U*_eq_	
O1	−0.0067 (2)	0.60204 (17)	0.30887 (18)	0.0406 (5)	
O2	0.1226 (2)	0.62774 (19)	0.53341 (19)	0.0494 (6)	
H2	0.1995	0.5993	0.5951	0.080*	
C1	0.2147 (3)	0.4753 (2)	0.4208 (2)	0.0376 (6)	
N1	−0.0869 (3)	0.8121 (2)	0.4675 (2)	0.0444 (6)	
H1	−0.0212	0.7516	0.4899	0.080*	
C10	0.1036 (3)	0.5752 (2)	0.4219 (3)	0.0361 (6)	
O3	0.5211 (3)	0.1947 (2)	0.4273 (2)	0.0614 (7)	
H3	0.5014	0.1663	0.3499	0.080*	
C2	0.2031 (3)	0.4185 (3)	0.2978 (3)	0.0421 (7)	
H2A	0.1256	0.4433	0.2149	0.080*	
C8	−0.2648 (3)	0.9398 (2)	0.3478 (3)	0.0364 (6)	
H8	−0.3401	0.9793	0.2727	0.080*	
C7	−0.1811 (3)	0.8415 (3)	0.3413 (3)	0.0461 (7)	
H7	−0.1880	0.8005	0.2611	0.080*	
C4	0.4199 (3)	0.2872 (3)	0.4201 (3)	0.0438 (7)	
C6	0.3317 (3)	0.4363 (3)	0.5424 (3)	0.0478 (7)	
H6	0.3420	0.4738	0.6253	0.080*	
C3	0.3048 (3)	0.3260 (3)	0.2973 (3)	0.0449 (7)	
H3A	0.2960	0.2896	0.2143	0.080*	
C5	0.4330 (4)	0.3435 (3)	0.5433 (3)	0.0545 (8)	
H5	0.5102	0.3183	0.6262	0.080*	
N2	−0.2226 (4)	0.9731 (3)	0.4821 (3)	0.0715 (9)	
C9	−0.1105 (4)	0.8937 (3)	0.5572 (3)	0.0519 (8)	
H9	−0.0586	0.8941	0.6528	0.080*	

### Atomic displacement parameters (Å^2^)

**Table:**

	*U*^11^	*U*^22^	*U*^33^	*U*^12^	*U*^13^	*U*^23^
O1	0.0403 (10)	0.0418 (10)	0.0347 (10)	0.0012 (7)	0.0099 (8)	0.0032 (7)
O2	0.0488 (11)	0.0549 (12)	0.0346 (10)	0.0151 (9)	0.0065 (8)	−0.0054 (8)
C1	0.0384 (13)	0.0386 (13)	0.0336 (13)	−0.0009 (10)	0.0121 (11)	−0.0003 (10)
N1	0.0458 (12)	0.0424 (12)	0.0412 (13)	0.0075 (10)	0.0137 (10)	0.0059 (10)
C10	0.0366 (13)	0.0371 (13)	0.0319 (13)	−0.0023 (10)	0.0108 (10)	0.0022 (9)
O3	0.0610 (13)	0.0710 (15)	0.0436 (12)	0.0277 (11)	0.0122 (10)	−0.0073 (10)
C2	0.0438 (14)	0.0463 (14)	0.0319 (13)	0.0046 (11)	0.0109 (11)	0.0005 (10)
C8	0.0331 (12)	0.0372 (12)	0.0323 (12)	0.0079 (10)	0.0065 (10)	0.0097 (10)
C7	0.0475 (15)	0.0476 (15)	0.0386 (14)	0.0003 (12)	0.0125 (12)	−0.0002 (12)
C4	0.0435 (14)	0.0466 (15)	0.0398 (14)	0.0068 (11)	0.0154 (12)	−0.0027 (11)
C6	0.0517 (16)	0.0551 (16)	0.0305 (13)	0.0116 (13)	0.0101 (12)	−0.0063 (11)
C3	0.0498 (15)	0.0503 (15)	0.0327 (13)	0.0049 (12)	0.0146 (12)	−0.0052 (11)
C5	0.0527 (16)	0.0673 (19)	0.0316 (14)	0.0177 (14)	0.0048 (12)	−0.0024 (13)
N2	0.075 (2)	0.0681 (19)	0.075 (2)	0.0103 (15)	0.0332 (17)	0.0024 (15)
C9	0.0572 (17)	0.0594 (18)	0.0343 (15)	0.0063 (14)	0.0137 (13)	0.0022 (12)

### Geometric parameters (Å, °)

**Table:**

O1—C10	1.269 (3)	C8—C7	1.330 (4)
O2—C10	1.249 (3)	C8—N2	1.358 (4)
O2—H2	0.8200	C8—H8	0.9300
C1—C6	1.387 (4)	C7—H7	0.9300
C1—C2	1.396 (4)	C4—C3	1.386 (4)
C1—C10	1.502 (4)	C4—C5	1.391 (4)
N1—C7	1.313 (4)	C6—C5	1.376 (4)
N1—C9	1.367 (4)	C6—H6	0.9300
N1—H1	0.8600	C3—H3A	0.9300
O3—C4	1.357 (3)	C5—H5	0.9300
O3—H3	0.8200	N2—C9	1.341 (4)
C2—C3	1.381 (4)	C9—H9	0.9300
C2—H2A	0.9300		
			
C10—O2—H2	109.5	N1—C7—H7	125.9
C6—C1—C2	118.0 (2)	C8—C7—H7	125.9
C6—C1—C10	120.8 (2)	O3—C4—C3	123.1 (2)
C2—C1—C10	121.2 (2)	O3—C4—C5	117.4 (2)
C7—N1—C9	108.7 (2)	C3—C4—C5	119.4 (2)
C7—N1—H1	125.7	C5—C6—C1	121.5 (2)
C9—N1—H1	125.7	C5—C6—H6	119.3
O2—C10—O1	122.8 (2)	C1—C6—H6	119.3
O2—C10—C1	118.9 (2)	C2—C3—C4	120.1 (2)
O1—C10—C1	118.3 (2)	C2—C3—H3A	119.9
C4—O3—H3	109.5	C4—C3—H3A	119.9
C3—C2—C1	121.0 (2)	C6—C5—C4	119.9 (3)
C3—C2—H2A	119.5	C6—C5—H5	120.0
C1—C2—H2A	119.5	C4—C5—H5	120.0
C7—C8—N2	108.9 (2)	C9—N2—C8	106.8 (3)
C7—C8—H8	125.6	N2—C9—N1	107.3 (3)
N2—C8—H8	125.6	N2—C9—H9	126.3
N1—C7—C8	108.2 (2)	N1—C9—H9	126.3

### Hydrogen-bond geometry (Å, °)

**Table:**

*D*—H···*A*	*D*—H	H···*A*	*D*···*A*	*D*—H···*A*
N1—H1···O1	0.86	2.527	3.057 (3)	120.73
N1—H1···O2	0.86	1.818	2.678 (3)	177.32
O3—H3···O1^i^	0.82	1.831	2.635 (3)	166.35
C8—H8···O1^ii^	0.93	1.886	2.748 (3)	153.18

Symmetry codes:  (i) −*x*+1/2, *y*−1/2, −*z*+1/2; (ii) −*x*−1/2, *y*+1/2, −*z*+1/2.
